# Limited Sensitivity of Stress Testing for Detecting Severe Coronary Artery Disease in Liver Transplant Candidates: A Retrospective Cohort Study

**DOI:** 10.7759/cureus.109801

**Published:** 2026-05-28

**Authors:** Carlos Huerta, William Giesing, Michael Mogg, Adil Khetani, Abdulmanan Abid, Hira Cheema, Candy Delgado, Bryan Barrientos, Laurette Mbuntum, Abul N Khan

**Affiliations:** 1 Internal Medicine, Methodist Health System, Dallas, USA; 2 Cardiovascular Disease, Methodist Health System, Dallas, USA

**Keywords:** cad: coronary artery disease, cardiac screening, cardiac stress test, diagnostic performance, liver cirrhosis (lc), orthotopic liver transplantation

## Abstract

Background: Cardiovascular disease is a leading cause of morbidity and mortality following liver transplantation. Current transplant evaluation protocols frequently rely on noninvasive stress testing to identify candidates at risk for obstructive coronary artery disease (CAD).

Aim: To evaluate the diagnostic performance of cardiac stress testing for detecting severe CAD in liver transplant candidates.

Materials and Methods: We conducted a retrospective cohort study of adult patients undergoing evaluation for orthotopic liver transplantation at a tertiary transplant center. Patients underwent pre-transplant cardiac assessment with stress testing, invasive coronary angiography, or both. Severe CAD was defined as ≥70% stenosis in a major epicardial coronary artery on angiography. The diagnostic performance of stress testing was assessed in patients who underwent both modalities.

Results: Among 637 transplant candidates, 599 (94.0%) underwent stress testing, and 240 (37.7%) underwent coronary angiography; 234 patients (36.7%) underwent both tests and were included in the diagnostic analysis. Severe CAD was identified in 26 patients (11.1%). Pharmacological stress testing demonstrated low sensitivity for detecting severe CAD (15.4%, 4/26; 95% confidence interval (CI): 4.4-34.9%) with specificity of 86.1% (179/208; 95% CI: 80.7-90.5%). The positive predictive value was 12.1% (4/33), and the negative predictive value was 89.1% (179/201). Notably, 22 of 26 patients (84.6%) with severe CAD had normal stress test results. Eleven patients (1.7%) ultimately underwent percutaneous coronary intervention (PCI), of whom 10 (90.9%) had normal stress tests, corresponding to approximately 55 stress tests performed per patient identified as requiring PCI.

Conclusion: In this cohort of liver transplant candidates, stress testing demonstrated poor sensitivity for detecting severe CAD. These findings highlight critical limitations of current cardiac screening strategies and suggest that reliance on stress testing alone may result in under-detection of clinically significant CAD. Alternative approaches, including coronary computed tomography angiography (CCTA), warrant prospective evaluation for pre-transplant cardiac risk stratification.

## Introduction

Cardiovascular disease is a leading cause of non-hepatic morbidity and mortality following liver transplantation, accounting for approximately 30-40% of early post-transplant deaths [1,2]. Comprehensive pre-transplant cardiovascular risk assessment is therefore essential to identify patients at risk for obstructive coronary artery disease (CAD) and to improve perioperative and long-term cardiovascular outcomes.

Patients with cirrhosis exhibit a distinct cardiovascular phenotype characterized by systemic vasodilation and increased cardiac output [3,4]. These hemodynamic changes contribute to cirrhotic cardiomyopathy, a syndrome defined by impaired contractile reserve and blunted responses to physiologic stress [5]. As a result, traditional preoperative cardiac testing may be difficult to interpret in this population, as reduced cardiac reserve can attenuate the hemodynamic demands needed to provoke ischemia. In particular, vasodilatory pharmacological agents may exert amplified and indiscriminate effects in the already vasodilated circulatory state of cirrhosis, reducing the differential coronary flow reserve necessary to identify obstructive lesions [6].

Current clinical practice guidelines recommend cardiovascular risk stratification for patients undergoing major noncardiac surgery, including liver transplantation [7]. However, specific evidence-based screening strategies for liver transplant candidates remain limited, and evaluation strategies vary across transplant centers [3,8]. In most settings, screening begins with noninvasive testing, most commonly transthoracic echocardiography and stress testing to evaluate for inducible myocardial ischemia. Patients with abnormal results or high clinical suspicion for CAD often proceed to invasive coronary angiography for definitive evaluation of the presence of obstructive disease.

Despite its widespread use, the diagnostic performance of stress testing in patients with cirrhosis remains unclear. Early studies by Donovan et al. demonstrated limited sensitivity of dobutamine stress echocardiography for detecting obstructive CAD in liver transplant candidates [9], findings that were later confirmed by Harinstein et al. in a larger cohort despite advances in imaging technology [10]. Nevertheless, stress testing continues to serve as a primary cardiac screening modality in many transplant programs. It is important to note that previous studies largely evaluated dobutamine-based protocols, which differ mechanistically from the vasodilatory pharmacological agents used in contemporary practice [6]. Given that regadenoson-based stress testing comprised the vast majority of our cohort, our findings are most directly applicable to this modality.

In this context, we aimed to evaluate the diagnostic performance of cardiac stress testing for detecting severe CAD in a large cohort of liver transplant candidates at a tertiary transplant center.

## Materials and methods

Study Design and Patient Population

We conducted a single-center retrospective cohort study of 637 adults who underwent orthotopic liver transplantation between January 1, 2017, and December 31, 2024. Consecutive patients referred for liver transplant evaluation were identified through our institutional transplant database, and relevant clinical data were extracted from the electronic medical record (EMR). Patients with incomplete pre-transplant cardiac workups were retained in the analysis if sufficient data were available to determine stress test results and/or angiographic findings. The study was approved by the WCG Institutional Review Board (IRB) (protocol #040.HEP.2025.A) and conducted in accordance with the Declaration of Helsinki. A waiver of informed consent was granted.

Cardiac Evaluation Protocol

Pre-transplant cardiac evaluation was performed at the discretion of the referring transplant hepatologist and consulting cardiologist. Stress testing modalities included both pharmacological and exercise-based stress testing with imaging (echocardiographic or nuclear). Invasive left heart catheterization (LHC) was performed at the discretion of the treating cardiologist based on overall clinical risk assessment, regardless of stress test result. The predominant stress testing modality was pharmacological stress testing with regadenoson (583/596, 97.8%), with a minority undergoing dobutamine stress testing (9, 1.5%), exercise stress testing (3, 0.5%), or adenosine stress testing (1, 0.2%). Of the 599 patients who underwent stress testing, 596 had their specific modality documented in the EMR. The remaining three patients had stress testing performed, but the specific modality was not recorded in the EMR. Six patients underwent LHC without previous stress testing and were excluded from the diagnostic performance analysis.

Definitions

Severe CAD was defined as ≥70% stenosis in at least one major epicardial coronary artery, as documented in coronary angiography reports. Stress test results were classified as normal or abnormal based on the interpreting cardiologist’s final report. Percutaneous coronary intervention (PCI) was performed at the discretion of the treating interventional cardiologist.

Diagnostic Performance Analysis

The primary analysis evaluated the diagnostic performance of stress testing for detecting severe CAD in the paired diagnostic cohort, defined as patients who underwent both stress testing and coronary angiography. Sensitivity, specificity, positive predictive value, and negative predictive value were calculated using standard 2×2 contingency table methods. Exact 95% confidence intervals (CIs) were calculated using the Clopper-Pearson method.

Statistical Analysis

Continuous variables are presented as mean and standard deviation (SD) or median with interquartile range (IQR), as appropriate. Categorical variables are presented as counts and percentages. Between-group comparisons were performed using independent-samples t-tests or Mann-Whitney U tests for continuous variables, and chi-square or Fisher's exact test for categorical variables, as appropriate. All analyses were performed using the R statistical software (version 4.3.2; R Foundation for Statistical Computing, Vienna, Austria).

## Results

Study Population

**Figure 1:**
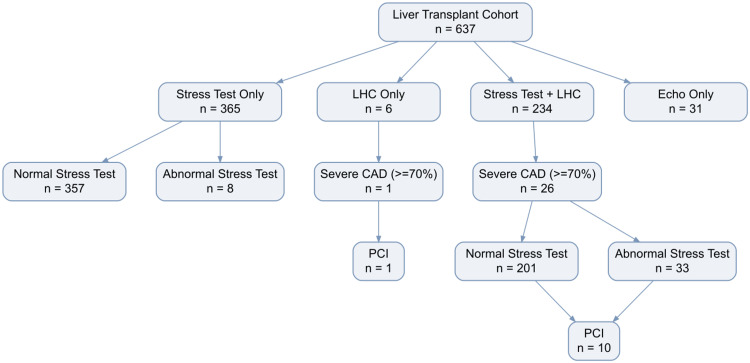
Flow Diagram of Cardiac Evaluation Pathway in Liver Transplant Candidates LHC: left heart catheterization; CAD: coronary artery disease; PCI: percutaneous coronary intervention; ECHO: echocardiography

A total of 637 liver transplant candidates were included in the study (Figure *1*). Baseline demographic and clinical characteristics are summarized in Table *1*. The mean age was 56.8 (10.5) years, and 417 (65.5%) were male. The most prevalent cardiovascular risk factors were hypertension (440, 69.1%), hyperlipidemia (257, 40.3%), and diabetes mellitus (256, 40.2%). The mean Model for End-Stage Liver Disease (MELD) score at transplant was 25.8 (8.9). The most common etiologies of liver disease were alcohol-associated liver disease (203, 31.9%) and metabolic dysfunction-associated steatohepatitis/nonalcoholic steatohepatitis (155, 24.3%).

**Table 1:** Baseline Demographic and Clinical Characteristics (N = 637) Categorical variables are presented as n (%). Continuous variables are presented as mean ± SD. Between-group comparisons were performed using independent-samples t-tests for continuous variables and chi-square tests for categorical variables. BMI: body mass index; CAD: coronary artery disease; CKD: chronic kidney disease; ESRD: end-stage renal disease; LHC: left heart catheterization; MELD: Model for End-Stage Liver Disease; SD: standard deviation

Characteristic	Overall (N=637)	LHC-based (N=240)	Non-invasive (N=397)	p-value (test statistic)
Age at transplant, mean (SD), years	56.8 (10.5)	61.9 (6.7)	53.7 (11.1)	<0.001 (t=10.31)
BMI, mean (SD), kg/m²	29.8 (6.4)	30.7 (6.0)	29.2 (6.6)	0.006 (t=2.78)
Sex				0.074 (χ² = 3.19)
Male	417 (65.5%)	156 (65.0%)	261 (65.7%)	
Female	220 (34.5%)	84 (35.0%)	136 (34.3%)	
Tobacco use				0.300 (χ² = 2.41)
Never smoker	358 (56.2%)	140 (58.3%)	218 (54.9%)	
Former smoker	264 (41.4%)	97 (40.4%)	167 (42.1%)	
Current smoker	15 (2.4%)	3 (1.2%)	12 (3.0%)	
Renal function				<0.001 (χ² = 21.85)
No CKD/ESRD	375 (58.9%)	114 (47.5%)	261 (65.7%)	
CKD	217 (34.1%)	101 (42.1%)	116 (29.2%)	
ESRD	45 (7.1%)	25 (10.4%)	20 (5.0%)	
Cardiovascular risk factors				
Hypertension	440 (69.1%)	207 (86.2%)	233 (58.7%)	<0.001 (χ² = 51.90)
Hyperlipidemia	257 (40.3%)	140 (58.3%)	117 (29.5%)	<0.001 (χ² = 50.58)
Diabetes mellitus	256 (40.2%)	188 (78.3%)	68 (17.1%)	<0.001 (χ² = 230.56)
Previous CAD	53 (8.3%)	48 (20.0%)	5 (1.3%)	<0.001 (χ² = 66.43)
Liver disease characteristics				
MELD score, mean (SD)	25.8 (8.9)	24.3 (8.5)	26.8 (9.1)	0.001 (t=3.45)

Of the 637 transplant candidates, 599 (94.0%) underwent stress testing and 240 (37.7%) underwent LHC. The median time from stress testing to transplantation was 150 days (IQR: 52-302), and from LHC to transplantation was 196 days (IQR: 61-413). The median interval between stress testing and coronary angiography was 37 days (IQR: 22-60 days). Stress testing was performed in 599/637 patients (94.0%), of whom 558 (93.2%) had normal results. Furthermore, 201 of 234 patients (85.9%) who proceeded to LHC had a normal stress test result, reflecting that LHC referral was driven by overall cardiovascular risk profile rather than stress test findings alone. Patients who underwent LHC were older (mean age 61.9 (6.7) vs. 53.7 (11.1) years, p<0.001) and had significantly higher rates of hypertension, diabetes mellitus, hyperlipidemia, chronic kidney disease, and previous known CAD compared to those who did not (all p<0.001), consistent with the selection of higher-risk individuals for invasive evaluation. The groups were otherwise similar with respect to markers of liver disease severity, including rates of ascites, variceal bleeding history, and tobacco use (all p>0.05). Among the 240 patients who underwent LHC, 117 (48.8%) had normal coronary arteries, 123 (51.2%) had any CAD, 27 (11.3%) had severe CAD (≥70% stenosis), and 11 (4.6%) underwent PCI.

Diagnostic Performance of Stress Testing

The paired diagnostic cohort included 234 patients who underwent both stress testing and coronary angiography. Severe CAD (≥70% stenosis) was identified in 26 of 234 patients (11.1%). Stress testing demonstrated poor sensitivity for detecting severe CAD (4/26, 15.4%; 95% CI: 4.4-34.9%) with moderate specificity (179/208, 86.1%; 95% CI: 80.7-90.5%). The positive predictive value was 12.1% (4/33; 95% CI: 2.6-31.2%), and the negative predictive value was 89.1% (179/201; 95% CI: 84.0-93.0%). Detailed diagnostic performance metrics are presented in Tables 2-3.

**Table 2:** 2×2 Contingency Table: Stress Test vs. Severe CAD on Angiography (Paired Cohort, n=234) Severe CAD was defined as ≥70% stenosis in a major epicardial coronary artery on coronary angiography. CAD: coronary artery disease

	Severe CAD on angiography		
	Present	Absent	Total
Stress test, abnormal	4	29	33
Stress test, normal	22	179	201
Total	26	208	234

**Table 3:** Diagnostic Performance of Stress Testing for Severe CAD (Paired Cohort, n=234) Severe CAD was defined as ≥70% stenosis in a major epicardial coronary artery on coronary angiography. Confidence intervals were calculated using the Clopper-Pearson exact method. CAD: coronary artery disease; CI: confidence interval; PCI: percutaneous coronary intervention

Diagnostic metric	Value (n=234)
Sensitivity	4/26 (15.4%; 95% CI: 4.4-34.9%)
Specificity	179/208 (86.1%; 95% CI: 80.7-90.5%)
Positive predictive value (PPV)	4/33 (12.1%; 95% CI: 2.6-31.2%)
Negative predictive value (NPV)	179/201 (89.1%; 95% CI: 84.0-93.0%)
False-negative rate	22/26 (84.6%) with severe CAD
Patients requiring PCI with a normal stress test	10/11 (90.9%)
Stress tests per PCI-requiring patient identified	~55

Notably, 22 of 26 patients (84.6%) with angiographically confirmed severe CAD had normal stress test results, indicating a high false-negative rate. Among the 11 patients who ultimately underwent PCI, 10 (90.9%) had normal pre-procedural stress tests. In total, 599 stress tests were performed across the cohort to identify 11 patients ultimately requiring coronary revascularization, corresponding to approximately 55 stress tests per patient identified as requiring PCI.

## Discussion

This retrospective cohort study suggests that cardiac stress testing may have limited effectiveness as a screening tool for liver transplant candidates, as most patients with severe CAD initially presented with normal stress test results. These findings underscore a critical limitation of stress testing as a frontline cardiac screening strategy in this population.

The limited diagnostic performance of stress testing in cirrhotic patients is likely multifactorial. Advanced cirrhosis is characterized by systemic vasodilation, reduced systemic vascular resistance, and compensatory increases in cardiac output, all of which may attenuate the ability to generate the relative myocardial ischemia required for a positive stress test [3,4]. Additionally, cirrhotic cardiomyopathy is associated with a blunted heart rate response and reduced exercise capacity, further limiting the sensitivity of exercise-based protocols [5]. Pharmacological vasodilators, including adenosine, dipyridamole, and regadenoson, may have diminished effect in this setting, as pre-existing systemic vasodilation reduces the coronary flow reserve gradient needed to produce detectable perfusion defects [5,6].

These findings are consistent with those of previous studies. Donovan et al. first reported limited sensitivity of dobutamine stress echocardiography in liver transplant candidates [9]. Harinstein et al. subsequently confirmed these findings in a larger, more contemporary cohort [10]. Taken together, the available evidence suggests that the diagnostic limitations of stress testing in cirrhosis are consistent and reproducible, with important implications for pre-transplant screening strategies.

A clinically important observation in the present study was that 10 of 11 patients (90.9%) who ultimately required PCI had normal pre-transplant stress tests. The low diagnostic yield, approximately 55 tests performed per actionable coronary lesion identified, raises concerns regarding the efficiency and utility of stress testing as a screening strategy in unselected liver transplant candidates.

Notably, patients who underwent non-invasive evaluation alone had a higher mean MELD score compared to those who underwent LHC-based evaluation (26.8 vs. 24.3, p=0.001). This counterintuitive finding suggests that the most critically ill candidates, who may have the greatest urgency for transplantation, are paradoxically less likely to undergo invasive cardiac evaluation. As a result, a subset of high-risk patients may proceed to transplantation with undetected obstructive CAD, representing a potential gap in current pre-transplant cardiac screening practices that warrants further investigation.

These findings highlight the potential utility of coronary computed tomography angiography (CCTA) as an alternative or complementary screening modality. CCTA provides a direct anatomic assessment of coronary stenosis and has demonstrated high sensitivity and negative predictive value for obstructive CAD in non-cirrhotic populations [11]. Several transplant centers have begun incorporating CCTA into pre-transplant evaluation protocols [12]. Notably, CAD has been detected in up to 36.8% of liver transplant candidates undergoing protocol coronary angiography, underscoring the high burden of undetected disease in this population [13]. That said, CCTA is not without limitations in this patient population: advanced liver disease is frequently associated with renal dysfunction and elevated coronary calcium burden, both of which may affect the feasibility and interpretability of CCTA in routine clinical practice. As the predominant modality in the present cohort was regadenoson-based stress testing, these findings and the limitations described above should be interpreted within that context when designing future prospective studies directly comparing stress testing and CCTA in liver transplant candidates.

This study has several limitations. As a single-center retrospective study, the findings may not be generalizable to other transplant programs with differing patient populations or evaluation protocols. The paired diagnostic cohort was not randomly selected; patients who underwent both stress testing and coronary angiography had higher cardiovascular risk profiles than the overall cohort, which may have influenced prevalence estimates and diagnostic performance. Accordingly, the reported diagnostic metrics were calculated within this paired cohort and should not be extrapolated to the entire transplant population. Furthermore, patients with previous coronary revascularization were not excluded from the analysis, as our aim was to reflect real-world pre-transplant cardiac evaluation practice. The inclusion of these patients may have influenced stress test interpretation and represents an additional limitation of this study. Data were extracted by the study team without independent blinded review, which represents an additional limitation of the retrospective design. Additionally, although the median interval between stress testing and coronary angiography was 37 days (IQR: 22-60 days), the interval between stress testing and transplantation (median 150, IQR: 52-302 days) may have allowed for progression of CAD, contributing to discordance between test results and angiographic findings. Finally, subgroup analyses by stress testing modality were not feasible due to limited sample sizes within each subgroup, restricting conclusions about the comparative performance of individual protocols.

## Conclusions

In this large single-center cohort of liver transplant candidates, cardiac stress testing demonstrated poor sensitivity and was not reliable for detecting severe CAD, with most patients with angiographically confirmed severe disease having falsely reassuring normal results. These findings raise important questions regarding the diagnostic limitations of current pre-transplant cardiac screening approaches and highlight the need for prospective evaluation of alternative strategies, such as CCTA, that may provide more reliable anatomic assessment of coronary disease in patients with cirrhosis. Prospective studies are needed to establish whether undetected severe CAD in this population translates into worse perioperative and long-term cardiovascular outcomes.
